# Evaluation of social media videos on weight-loss surgery and GLP-1 agonists: a content quality and reliability analysis

**DOI:** 10.1007/s00464-025-11938-4

**Published:** 2025-07-09

**Authors:** Subin Lee, Nisha Narula, Monika Weglarz, Jenny Lee, Danbee Kim

**Affiliations:** 1 Rutgers New Jersey Medical School, Newark, NJ USA; 2 Rutgers New Jersey Medical School, Department of Surgery, 185 South Orange Avenue, Newark, NJ 07103 USA

**Keywords:** Obesity, Social media, GLP-1, Bariatric surgery, Sleeve gastrectomy

## Abstract

**Background:**

Social media platforms have become a leading arena for disseminating health knowledge. Information on weight loss is no exception; health, fitness and nutrition are within the 5 most searched categories in 2023. Our study investigates the quality of information on TikTok and Instagram regarding the two most popular weight loss treatments: glucagon-like-peptide-1 (GLP-1) agonist and sleeve gastrectomy.

**Methods:**

TikTok and Instagram were queried for contents about GLP-1 agonists or sleeve gastrectomy. The first 50 videos from each category were evaluated by two reviewers. The videos were divided into four categories: Instagram GLP-1, Instagram weight loss surgery (WLS), TikTok GLP-1, and TikTok WLS. The quality of the contents was assessed using the DISCERN score.

**Results:**

200 videos were collected for analysis. 31.5% were created by medical professionals and 68.5% were from non-medical professionals. More videos from Instagram regarding GLP-1 agonists followed by videos from TikTok regarding GLP-1 agonists were made by medical professionals compared to videos about surgery (*p* = 0.038). The average number of likes overall was 67,024 and saves or shares 6,305. The greatest number of likes were in the TikTok surgery group (*p* = 0.004) and the most saves or shares were in the Instagram surgery group (*p* < 0.001). DISCERN scores were low for all four categories, overall 28.0 out of 80. They were highest in the Instagram GLP-1 agonist group (*p* < 0.001).

**Conclusions:**

Despite the popularity of TikTok and Instagram, the quality of the health contents, as measured by DISCERN score, on these platforms remains poor. Most are created by non-medical professionals. Healthcare professionals should encourage patients to remain vigilant and educate them on misinformation on social media. This may also represent an opportunity for healthcare providers to generate their own social media content to ensure reliability of information as well as to direct patients to reliable social media content.

Obesity rates in the United States (US) remain high, affecting approximately 40% of the adult population. Meanwhile, the percentage of those with severe obesity, defined as body mass index (BMI) ≥ 40, continues to rise [1]. The ongoing obesity epidemic poses a threat to both the individual’s health as well as to the overall healthcare system. People who are overweight or obese have increased risk of morbidity from other chronic diseases such as hypertension, diabetes, sleep apnea, and coronary disease and have a decreased life expectancy [2, 3]. The healthcare cost is also substantial, reaching nearly $173 billion in 2019 alone [4]. Moreover, obesity rates are projected to increase, and by 2030, nearly 1 out of every 2 adults in the US are anticipated to be obese. Accelerated efforts are needed to improve our strategies in achieving weight loss.

The use of social media for health promotion has been gaining interest. As of 2023, TikTok and Instagram have surpassed Google as the preferred search engine for those aged 18–24 [5]. As such, it’s not surprising that social media influences health behaviors both positively and negatively. Engagement in social media has been associated with changes in the individual’s perception of body image, disordered eating behaviors, and riskier health behaviors such as substance use and gambling [6–8]. On the other hand, social media use can prompt social connectedness, gather peer support, and boost physical activity [9–11].

Though widely used, the medical information portrayed in these social media platforms is of varying quality. One study that analyzed the top 100 TikTok videos on medication abortion showed an accuracy of 89.2%, while others have found TikTok videos on topics such as attention deficit/hyperactivity disorder, irritable bowel syndrome, gender affirmation surgeries, and abdominoplasties to be unreliable. For instance, some of these studies analyzed the quality of social media videos on irritable bowel syndrome and aesthetic procedures such as breast augmentation and rhinoplasty. Using the modified DISCERN score, which is a 5-point Likert scale grading the quality of health information, the results of these studies showed that the quality of these video contents were poor, with scores ranging between 1.25 and 1.9 out of 5 [12–16]. However, despite the abundance of health information on weight loss treatments in social media, studies assessing the quality of these contents are scarce. The goal of this study is to analyze the accuracy of TikTok and Instagram videos on two popular weight loss treatments, GLP-1 agonists and sleeve gastrectomy.

## Materials and methods

TikTok and Instagram were searched for two different weight loss treatments, GLP-1 agonist or sleeve gastrectomy. Hashtags consisting of phrases “glp1,” “glp-1,” “semaglutide,” and “Ozempic” were used for the former and “gastric sleeve,” “gastric sleeve surgery,” “vsg,” “vsg community,” and “weight loss surgery” for the latter. The study design was reviewed and approved by the Institutional Review Board at Rutgers University.

Fifty videos from each of the four categories that met the inclusion criteria were selected. These four categories are TikTok videos on GLP-1 agonist, TikTok videos on sleeve gastrectomy, Instagram videos on GLP-1 agonist, and Instagram videos on sleeve gastrectomy. Videos that were in languages other than English, with sponsored contents, or with no audio or visual explanations were excluded. Descriptive characteristics for each of the videos were recorded, including content creator (medical provider vs. non-medical provider), number of likes, number of saves or shares, and the general perspectives of the videos, and the general perspectives of the comments, both of which were categorized as positive, negative or neutral. The quality of the video was assessed with the DISCERN score, a validated tool grading the quality of consumer health information [17]. The tool consists of 16 questions, each rated on a scale of 1 to 5. The higher the score, the higher the quality and reliability of the information analyzed. Two independent reviewers, J.L. and M.W., interpreted the video content and scored the quality of each video. One reviewer is a general surgery resident and the other a medical student who have both been well-versed in the use of DISCERN score and supervised by a fellowship-trained bariatric surgeon. The DISCERN score was averaged between the two reviewers.

SPSS (IBM Corp released 2022 IBM SPSS Statistics, version 29.0. Armonk, NY: IBM Corp) was used for statistical analysis. Results are presented as means with standard deviation or percentages with numbers. The Chi-squared test, Kruskal–Wallis test, and Mann–Whitney U test were used to compare the groups. Multivariate analysis with logistic regression was used to determine the effects of the variables on DISCERN score using variables that were significant on univariate analysis. A *p*-value of < 0.05 was considered significant, and two-tailed tests were used.

## Results

A total of 200 videos were included, 50 in each group categorized as Instagram GLP-1 agonist, Instagram sleeve gastrectomy (SG), TikTok GLP-1 agonist, and TikTok SG. Table 1 describes the characteristics of the videos. There was a total of 67,024 likes and 6,305 saves or shares. Most of the videos were created by non-medical providers, rather than medical providers. The average DISCERN score was 28.0 out of 80. The contents of the videos were viewed as positive for the majority of the videos but most of the comments on videos were neutral. Comments were considered neutral if they were irrelevant to the content of the videos, composed mainly of questions such as “how much does sleeve gastrectomy surgery cost?,” or contained both positive and negative information, for instance, “GLP1 agonists are effective for weight loss but are known to have gastrointestinal side effects.

**Table 1:** Overall statistics

	Total N = 200 (except comments, where N = 189)
Medical provider posted	31.5% (63)
Video	
Positive	51.5% (103)
Neutral	30.0% (60)
Negative	18.5% (37)
Comments	
Positive	45.0% (85)
Neutral	45.5% (86)
Negative	9.5% (18)
Likes	67024 (321431)
Saves or shares	6305 (29276)
DISCERN Score	28.0 (8.4)

Of the four categories, the Instagram GLP-1 agonists group had the highest percentage of medical provider-produced videos. These videos also had the highest DISCERN score at 33.1 (*p* < 0.001). On the other hand, the TikTok videos on sleeve gastrectomy garnered the most likes (*p* = 0.004), while Instagram videos on sleeve gastrectomy had the highest number of saves or shares (*p* < 0.001) (Table 2).

**Table 2:** Comparison based on social media platform and posting regarding medicine or surgery

	Instagram GLP-1 N = 50	Instagram SG N = 50	TikTok GLP-1 N = 50	TikTok SG N = 50	p-value
Medical provider posted	46.0% (23)	24.0% (12)	34.0% (17)	22.0% (11)	0.038
Video					0.262
Positive	48.0% (24)	66.0% (33)	48.0% (24)	44.0% (22)	
Neutral	32.0% (16)	26.0% (13)	28.0% (14)	34.0% (17)	
Negative	20.0% (10)	8.0% (4)	24.0% (12)	22.0% (11)	
Comments (N = 189)					0.026
Positive	50.0% (24)	38.6% (17)	34.0% (17)	57.4% (27)	
Neutral	35.4% (17)	45.5% (20)	62.0% (31)	38.3% (18)	
Negative	14.6% (7)	15.9% (7)	4.0% (2)	4.3% (2)	
Likes	7943 (17225)	87432 (276691)	64216 (171751)	108504 (554523)	0.004
Saves or shares	2447 (6407)	10895 (41202)	4394 (8666)	7485 (40314)	< 0.001
DISCERN Score	33.1 (8.7)	25.5 (7.0)	25.6 (8.6)	27.9 (7.2)	< 0.001

*GLP-1* glucagon-like peptide-1, *SG* sleeve gastrectomy

Univariate analysis looking at factors associated with DISCERN score revealed that TikTok videos and videos on sleeve gastrectomy scored lower. Additionally, videos created by non-medical providers, with the most number of likes, and generally rated as containing positive contents were associated with lower DISCERN scores (Table 3).

**Table 3:** Associations with high or low DISCERN score

	Low score % and N or mean and SD	High score % and N or mean and SD	*p*-value
Social media modality			0.046
Instagram	43.9% (50)	58.1% (50)	
TikTok	56.1% (64)	41.9% (36)	
Medication/surgery			0.010
GLP-1	42.1% (48)	60.5% (52)	
Surgery	57.9% (66)	39.5% (34)	
Medical provider			< 0.001
Medical	12.3% (14)	57.0% (49)	
Nonmedical	87.7% (100)	43.0% (37)	
Video			0.013
Positive	60.5% (69)	39.5% (34)	
Neutral	24.6% (28)	37.2% (32)	
Negative	14.9% (17)	23.3% (20)	
Comments (N = 189)			0.66
Positive	47.7% (53)	41.0% (32)	
Neutral	43.2% (48)	48.7% (38)	
Negative	9.0% (10)	10.3% (8)	
Likes	97797 (412835)	26231 (111164)	0.023
Saves or shares	8857 (38132)	2922 (7296)	0.49

*GLP-1* glucagon-like peptide-1

On multivariate analysis, postings from non-medical providers were correlated with lower DISCERN score. A neutral or negative video was associated with a higher DISCERN score. Social media platform, contents on GLP-1 agonists versus surgery, and number of likes were not associated with statistically significant higher DISCERN scores (Table 4).

**Table 4:** Multivariate analysis for factors associated with high DISCERN score

Variable	Odds Ratio (OR)	*p*-value	95% CI for OR
Social media platform	0.53	0.064	0.27–1.04
Surgery related	0.61	0.15	0.32–1.19
Nonmedical provider	0.117	< 0.001	0.056–0.25
Likes	1.0	0.11	1.0–1.0
Neutral/negative video	2.27	0.017	1.16–4.45

## Discussion

Topics on body image and weight loss are becoming more and more prevalent on social media platforms. It provides users easy access to a variety of health information and enables them to readily communicate and share this information with other users. In this regard, social media use has the potential to provide valuable social interaction and peer support and change people’s health behaviors [18, 19]. The objective of our study was to assess the quality of TikTok and Instagram videos on the two most common weight loss treatment modalities, glucagon-like-peptide-1 agonists and sleeve gastrectomy, and determine factors associated with improved video accuracy.

The ability for people to readily create, post and share contents through social media often allows for misleading or inaccurate information to be disseminated to the public. Recently, we have seen a significant rise in the popularity of the diabetes drug Ozempic®, a GLP-1 agonist, as many celebrities and influencers claimed its weight loss benefits on social media platforms. This is in spite of the fact that the Federal Drug Administration (FDA) has never approved Ozempic for chronic weight management. [20] Similarly, a study by Samuel et al. looking at TikTok videos with #weightloss showed that less than a fifth of the videos were informational (19%) with the majority of these videos posted by consumers (93%) [21].

Despite the growing number of videos on TikTok and Instagram on obesity and weight loss, studies that assess the accuracy of these videos remain scarce. To date, our study is the first to objectively analyze the quality of social media videos on both medical and surgical treatment for weight loss. The overall quality of the 200 videos included in our study was low, with a DISCERN score of 28 out of 80, which is classified as a poor score. As expected, the majority of these videos were created by non-medical providers at 68.5%, and on multivariate analysis, these were associated with lower DISCERN scores compared to videos created by medical providers.

Similar to our findings, previous studies evaluating the contents of bariatric surgery videos on the Internet and social media platforms such as TikTok and YouTube have shown that the information obtained is, in general, of low quality [22–25]. For instance, the study by Lahooti et al. looking at 150 TikTok videos on three different weight loss procedures, vertical sleeve gastrectomy, gastric bypass and endoscopic sleeve gastroplasty found that the mean DISCERN score was 31.57. The study also noted higher scores were associated with videos made by physicians (*p* < 0.001) and those involving educational content (*p* < 0.001). On the other hand, popular videos with the most likes and shares were associated with lower DISCERN scores [25].

In addition to the overall inaccuracies of social media videos on weight loss treatments, certain DISCERN questions merit special attention as they highlight the alarming health misinformation that is readily available to patients. Questions 4 asks “Is it clear what sources of information were used to compile the publication (other than the author or producer)?, question 7 “Does it provide details of additional sources of support and information?”, and questions 10 & 11 “Does it describe the benefits/risks of each treatment?” Some of the Tiktok and Instagram videos from non-medical providers that were included in our study promoted one treatment option over another or went on to promote their fitness practice or holistic medicine instead while dismissing the use of GLP1 agonists or sleeve gastrectomy. These contents created to “educate” and “inform” the audience had unclear or false basis for their claims. Similar findings were reports in the study by Lahooti et al. showing us that we must remain vigilant to the biases that patients were influenced to when we encounter patients in the healthcare settings.

These findings point to areas where healthcare professionals can intervene on to improve the accuracy of health information that is being portrayed on social media. Efforts to better target social media users and create content that are more relatable and appealing to them are needed.

This study has several limitations. Tiktok and Instagram algorithms that provide unique experiences to users based on an individual’s viewing history lend themselves to variability in the resultant video prioritization. This, in turn, may affect the generalizability of our results. Additionally, the hashtags of interest used in our study is varied but not all encompassing, which limits the types of videos that were selected for analysis. Lastly, not all GLP-1 agonists are FDA approved for weight loss, however, the hashtags we used were broad which could have impacted the information regarding weight loss in these selected videos.

Despite the limitations, however, this study adds to the literature because it highlights the misinformation that is readily available to consumers on important health topics, raising concerns for its potential impact on patients. The focus on popular weight loss treatments is also novel and less well-studied.

With the rising popularity of social media platforms such as TikTok and Instagram, more users are turning to these outlets as a major source of news and information [26]. Information on health and wellness is no exception. Our study highlights the importance of increasing medical provider participation in promoting accurate health information on social media. Future studies are needed to better characterize the various factors that can contribute to producing higher quality videos while garnering better views. Interventions should include generating more accurate social media content and also identifying current accurate social media information and directing patients to reputable postings.
